# Disordered Eating Attitudes and Food Choice Motives Among Individuals Who Follow a Vegan Diet in Brazil

**DOI:** 10.1001/jamanetworkopen.2023.21065

**Published:** 2023-06-29

**Authors:** Bruna Caruso Mazzolani, Fabiana Infante Smaira, Gabriel P. Esteves, Martin Hindermann Santini, Alice Erwig Leitão, Heloísa C. Santo André, Bruno Gualano, Hamilton Roschel

**Affiliations:** 1 Applied Physiology and Nutrition Research Group, University of São Paulo, Sao Paulo, Brazil; 2 Food Research Center, University of São Paulo, Sao Paulo, Brazil; 3 School of Applied Sciences, State University of Campinas, Limeira, Brazil

## Abstract

**Importance:**

It remains controversial whether adhering to a vegan diet may be associated with a higher prevalence of disordered eating. Also, main food choice motives and their association with disordered eating in this population are still unknown.

**Objective:**

To determine the association between disordered eating attitudes and food choice motives of individuals who follow a vegan diet.

**Design, Setting, and Participants:**

This is a cross-sectional online survey conducted between September 2021 and January 2023. Individuals of both sexes, aged 18 years or older, following a vegan diet for at least 6 months, currently living in Brazil were recruited through advertisements on social media.

**Exposure:**

Adherence to a vegan diet and food choice motives.

**Main Outcomes and Measures:**

Disordered eating attitudes and food choice motives.

**Results:**

Nine hundred seventy-one participants completed the online survey. Median (IQR) age and BMI of participants was 29 (24-36) years and 22.6 (20.3-24.9), respectively, and 800 participants (82.4%) were female. Most participants (908 respondents [94%]) were categorized with the lowest level of disturbed eating attitudes. “Need and hunger,” “liking,” “health,” “habits,” and “natural concerns” were the most important food choice motives in this population, while “affect regulation,” “social norms,” and “social image” were the less important ones. Adjusted models showed that “liking,” “need and hunger,” and “health” were associated with lower levels of disordered eating attitudes, while “price,” “pleasure,” “sociability,” “traditional eating,” “visual appeal,” “social norms,” “social image,” “weight control,” and “affect regulation” were associated with higher levels of disordered eating attitudes.

**Conclusions and Relevance:**

In this cross-sectional study, as opposed to previous suggestions, levels of disordered eating were very low among vegans, although certain food choice motives were associated with disordered eating attitudes. Understanding the motivations of adhering to diets that may impose restrictions, which include vegan diets, may help tailor interventions focused on promoting healthy eating and preventing or treating disordered eating.

## Key Points

**Question:**

Is there an association between disordered eating attitudes and food choice motives among vegan dieters?

**Findings:**

In this cross-sectional study including 971 individuals who follow a vegan diet, 94% were categorized with the lowest level of disordered eating attitudes, with only 0.6% of participants being associated with disordered eating attitudes. Some food choice motives were associated with disordered eating attitudes in this sample.

**Meaning:**

Understanding the motivations of adhering to diets that may impose restrictions, which include vegan diets, may help tailor interventions focused on promoting healthy eating and preventing or treating disordered eating.

## Teaser

This cross-sectional study evaluates disordered eating attitudes and food choice motives of individuals who follow a vegan diet in Brazil.

## Introduction

Veganism has increased across the Western world in the last decade.^1,2^ The annual international Veganuary campaign, which encourages a temporary switch to a vegan diet, reported an increase in sign-ups from 3300 in 2013 to over 629 000 in 2022.^3^

An important concern regarding vegan diets is that they may be associated with a higher prevalence of eating disorder symptoms, as they impose dietary restrictions.^4,5,6,7,8^ In this context, veganism could be used to legitimize the rejection of food and social situations involving eating, consequently masking disordered eating attitudes by facilitating continued restriction. This may be the case when the switch to a vegan diet is motivated by the pursuit of weight loss and the exclusion of foods from the diet.^4,7^ In fact, a recent systematic review showed that vegetarianism (including veganism) appears to be associated with eating disorders.^4^ Also, some food choice motives (eg, weight control and affect regulation) may be associated with disordered eating attitudes in this population.^9,10^ Nonetheless, the association between motivations to follow a vegan diet and eating disorder symptoms remains controversial in the literature.^11,12^

The reasons for shifting and adhering to a vegan diet are various, such as religious and ethical beliefs, environmental concerns, cultural and social values, as well as health-related aspects.^13^ Gathering knowledge on eating motivation is key to understanding human behavior.^14^ Multiple reasons may explain eating choices, which are part of a complex interaction involving food characteristics, as well as biological, anthropological, psychological, cultural, and socioeconomic factors.^15,16^ Moreover, food choice motives have been shown to vary according to sex, body mass index (BMI), and socioeconomic status.^17,18,19^ However, it is still unknown how these motives are presented in individuals who decide to follow a vegan diet. Shedding light on this topic could allow us to better understand the motivations behind adhering to a vegan diet, as well as whether and how it is associated with disordered eating in this population. Therefore, the aims of the present study are to identify and associate disordered eating attitudes and food choice motives of individuals who follow a vegan diet.

## Methods

### Study Design and Participants

The Vegan Eating Habits and Nutritional Evaluation Survey (VEGAN-EatS) was a cross-sectional survey conducted between September 2021 and January 2023, aimed at gathering knowledge on multiple aspects of dietary behaviors in vegans. Participants were recruited through advertisements on social media sites (Facebook, WhatsApp, Instagram, and Twitter). Inclusion criteria were as follows: individuals of both sexes, aged 18 years or older, following a vegan diet for at least 6 months, currently living in Brazil, with the ability to read, and with internet access.

All participants completed an online survey on the Google Forms platform (Google LLC), which enquired about their demographic, socioeconomic, and anthropometric characteristics, lifestyle, disordered eating attitudes, and food choice motives. This study was approved by the local ethical committee (Certificate of Presentation of Ethical Appreciation) and was conducted in accordance with the Helsinki declaration^20^. An approved informed consent form was signed digitally by all participants before initiating the survey. This report follows the Strengthening the Reporting of Observational Studies in Epidemiology (STROBE) reporting guideline for cross-sectional studies.

### Evaluation Tool

The online survey included questions categorized into the following sections: (1) participant characteristics and lifestyle (age, educational level, income, smoking, drinking, and exercise habits); (2) anthropometric data (self-reported weight and height); and diet-related questions (duration of adherence to a vegan diet and motivations to shift toward a vegan diet). Income was categorized according to the Brazilian Institute of Geography and Statistics in income levels of A (≥US $4200), B (US $1350 to $4200), C (US $550 to $1350) and D/E (≤US $550).^21^

Subsequently, participants filled out 2 inventories. The first was the short version of the Disordered Eating Attitude Scale (DEAS) that evaluates the presence of disordered eating attitudes.^22^ It contains 17 questions and the final score ranges from 17 to 75 points. Individuals are categorized according to their score in the following categories: below 44.6; 44.6 to 49.9; 49.9 to 55.3; 55.3 to 60.7; 60.7 to 66.1; 66.1 to 71.4; and 71.4 to 76.8, with each successive category representing more dysfunctional eating attitudes. Scores of 66.1 or higher identified individuals with disordered eating attitudes.^22^ The second was the Brazilian Portuguese version of The Eating Motivation Survey (TEMS)^23^ that evaluates food choice motives (ie, “liking,” “health,” “natural concerns,” “need and hunger,” “habits,” “pleasure,” “convenience,” “weight control,” “sociability,” “traditional eating,” “price,” “visual appeal,” “affect regulation,” “social norms” and “social image”). This instrument comprises 45 questions preceded by the phrase “I eat what I eat…” and responses are given in a 5-point scale, from 1 (“never”) to 5 (“always”).

### Missing Data

Due to important missingness (33%) in anthropometric data (ie, body mass and height), individuals with and without these missing data were compared to visualize whether possible associations between missingness and important variables, such as DEAS score, were present (eTable in Supplement 1). There were only subtle differences between subgroups across all variables. However, given that body mass was a potential confounder (see subsequent Statistical Analysis section), we imputed this variable through multiple imputation,^24^ using the mice package in R,^25^ set to 5 iterations and the classification and regression trees method. A matrix of predictors was used to impute missing body mass data; the predictors selected were variables present in the main models, namely age, sex, income, exercise habits, motivation to shift to a vegan diet, and DEAS and TEMS scores. A comparison between original and imputed values is available in eFigure 1 in Supplement 1.

### Statistical Analysis

Descriptive data are presented as median and IQR for continuous variables and absolute and relative frequency (number and percentage) for categorical variables. Associations between food choice motives (independent variables) and disordered eating attitude scores (dependent variable) were individually (one model for each motive) tested using multivariable linear regression models. A directed acyclic graph was constructed to identify confounders of the possible outcomes of food choice motives on disordered eating attitudes, considering expert knowledge and current evidence from the literature (eFigure 2 in Supplement 1). All models were adjusted for the identified confounders, namely age, sex, body weight, income, exercise habits, and motivation to shift to a vegan diet. Assumptions of linear regression (linearity, normality of residuals, and so forth) were checked using appropriate visualizations.

These models were carried in all 5 imputed data sets, and results from models were pooled using the methods proposed by Rubin.^26^ As multiple regression models were used, *P* values were corrected for the false discovery rate using the Benjamini-Hochberg procedure.^27^ Pooled model results are presented as the unstandardized beta coefficient of each food choice motive and its corresponding 95% CI (ie, the average estimated change in disordered eating attitude score with each 1 unit increase in the respective food choice motive). A complete case analysis (ie, conducted in the subset of individuals with fully available data) is also present in eFigure 3 in Supplement 1. All analyses and visualizations were performed using R version 4.2.2 (R Project for Statistical Computing)^28^ and RStudio version 2022.12.0 (Posit Software, PBC), using the dplyr,^29^ ggplot2,^30^ and stats^28^ packages for data cleaning, visualizations, and linear models, respectively. The level of significance was set a priori at *P* ≤ .05.

## Results

**Figure 1.**
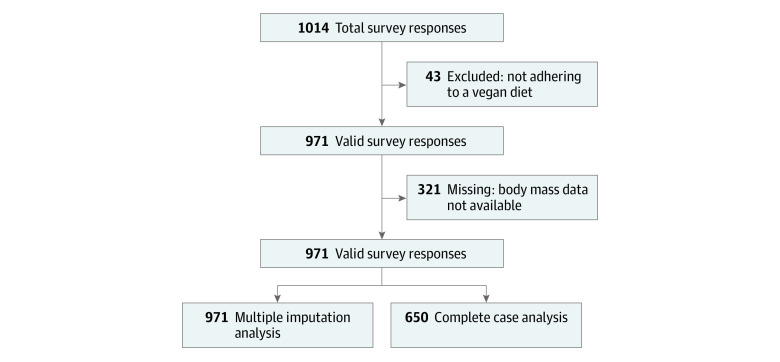
Study Flowchart

One thousand and fourteen participants completed the online survey; however, 43 were excluded for reportedly not following a vegan diet. Thus, data of 971 confirmed vegan dieters were used to describe sample characteristics and lifestyle, disordered eating attitudes and food choice motives (Figure 1). Linear models using imputed body mass values included 971 individuals, and a complete case analysis included 650 individuals (eFigure 3 in Supplement 1).

The median (IQR) age of participants was 29 (24-36) years, and the median (IQR) BMI of participants was 22.6 (20.3-24.9) (BMI is calculated as weight in kilograms divided by height in meters squared). The largest groups were female (800 respondents [82.4%]), had a postgraduate educational level (354 participants [36%]), belonged to socioeconomic class B (359 participants [37%]), reported no alcohol consumption (405 participants [42%]) or smoking habits (887 participants [91%]), exercised 3 to 4 hours per week (219 participants [23%]) and adhered to a vegan diet for 5 or more years (280 participants [29%]) (Table 1). The most cited motivations to adhere to a vegan diet were “ethics and animal rights” (587 participants [60%]), followed by “environment” (115 participants [12%]), “health reasons” (103 participants [11%]), and “life philosophy” (111 participants [11%]) (Table 1).

**Table 1.** Demographic, Anthropometric, and Lifestyle Characteristics

Characteristic	Participants, No. (%)		
	Overall (N = 971)	Female (n = 800)	Male (n = 171)
Age, median (IQR)	29 (24-36)	29 (24-36)	32 (26-36)
Body weight (kg), median (IQR)	61 (54-70)	59 (53-66)	73 (65-82)
Height (cm), median (IQR)	165 (160-170)	163 (159-168)	175 (170-180)
Body mass index, median (IQR)^a^	22.6 (20.3-24.9)	22.1 (20.1-24.5)	23.9 (22.5-26.3)
Educational level			
Postgraduate	354 (36)	300 (38)	54 (32)
College education or technician, complete	289 (30)	228 (28)	61 (36)
Undergoing college or technician education	222 (23)	176 (22)	46 (27)
High school, completed	75 (8)	68 (9)	7 (4)
High school, incomplete	22 (2)	22 (3)	0
Elementary school, completed	3 (<1)	1 (<1)	2 (1)
Elementary school, incomplete	5 (1)	4 (1)	1 (<1)
Other	1 (<1)	1 (<1)	0
Socioeconomic class^b^			
Class D/E	310 (32)	263 (33)	47 (27)
Class C	252 (26)	208 (26)	44 (26)
Class B	359 (37)	293 (37)	66 (38)
Class A	50 (5)	36 (5)	14 (8)
Smoking status	94 (10)	69 (89)	25 (15)
Alcohol consumption			
No alcohol consumption	405 (42)	335 (42)	70 (41)
Once to twice/mo	180 (19)	155 (19)	25 (15)
Twice to 4 times/mo	273 (28)	217 (27)	56 (33)
Twice to 3 times/wk	100 (10)	83 (10)	17 (10)
Four or more times/wk	13 (1)	10 (1)	3 (2)
Habitual physical exercise			
Does not exercise	194 (20)	168 (21)	26 (15)
1-2 h/wk	211 (22)	185 (23)	26 (15)
3-4 h/wk	219 (23)	181 (23)	38 (22)
5-6 h/wk	204 (21)	165 (21)	39 (23)
7 h/w or more	143 (15)	101 (13)	42 (25)
How long adhering to a vegan diet			
Less than 1 y	135 (14)	106 (13)	29 (17)
1 to 2 y	224 (23)	183 (23)	41 (24)
2 to 3 y	183 (19)	153 (19)	30 (18)
3 to 4 y	149 (15)	130 (16)	19 (11)
5 or more y	280 (29)	228 (28)	52 (30)
Motivation for adhering to a vegan diet			
Ethics and animal rights	587 (60)	510 (64)	77 (45)
Health reasons	103 (11)	79 (10)	24 (14)
Environment	115 (12)	97 (12)	18 (11)
Politics	41 (4)	30 (4)	11 (6)
Life philosophy	111 (11)	75 (9)	36 (21)
Medical restrictions	5 (1)	5 (1)	0
Religion	2 (<1)	1 (<1)	1 (1)
Sports performance	7 (1)	3 (<1)	4 (2)

^a^ Body mass index is calculated as weight in kilograms divided by height in meters squared.

^b^ Income was categorized according to the Brazilian Institute of Geography and Statistics in income levels of A (≥US $4200), B (US $1350 to $4200), C (US $550 to $1350) and D/E (≤US $550).^21^

**Figure 2.**
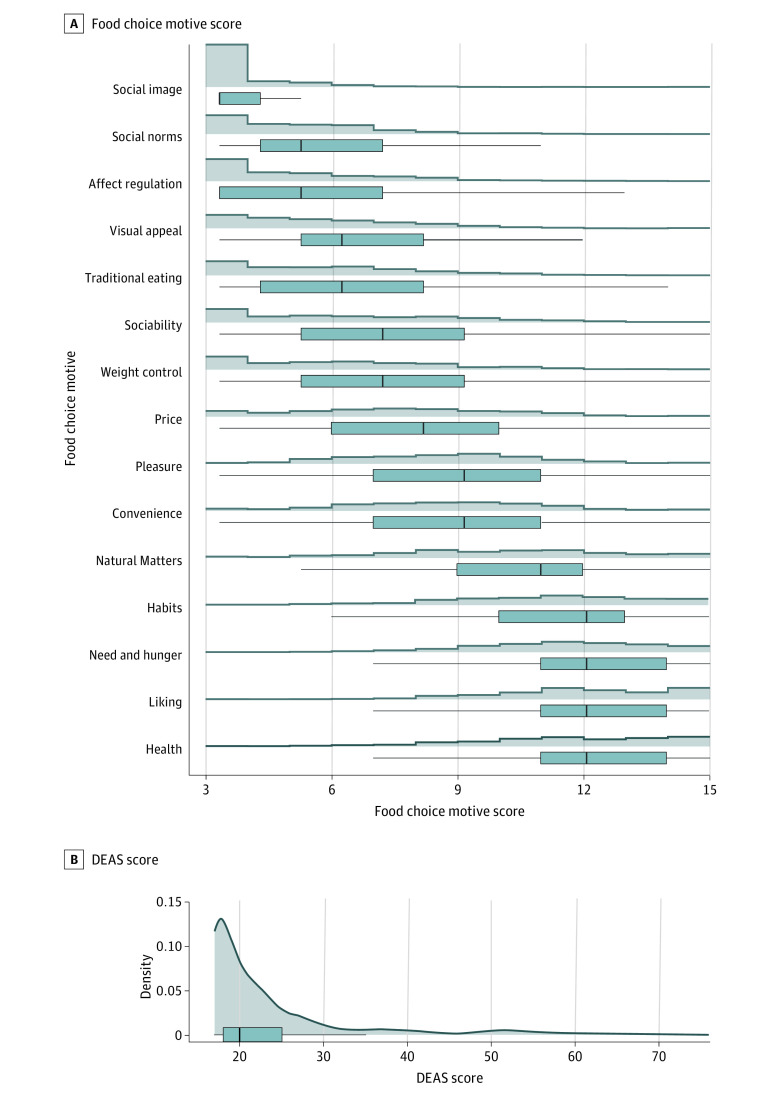
Distribution of Food Choice Motives and DEAS Scores in Individuals That Adhere to a Vegan Diet Panel A, distribution of food choice motives scores shown by histograms and traditional box and whisker plots. Panel B, distribution of DEAS scores shown by density curve and traditional box and whisker plot. DEAS indicates disordered eating attitude scale.

Median (IQR) DEAS score was 20 (18-25), with most participants (908 respondents [94%]) being categorized with the lowest level of disturbed eating attitudes.^22^ Most importantly, only 0.6% of our sample (1 participant) was identified with disordered eating attitudes. “Need and hunger,” “liking,” “health,” “habits,” and “natural concerns” were the most important (ie, presented higher scores) food choice motives in this population, while “affect regulation,” “social norms,” and “social image” were the least important ones (Table 2 and Figure 2).

**Table 2.** Food Choice Motives and Disordered Eating Attitudes

Attitude	Participants, median (IQR)		
	Overall (N = 971)	Female (n = 800)	Male (n = 171)
Food choice motives^a^			
Liking	12.0 (11.0-14.0)	12.0 (11.0-14.0)	12.0 (11.0-14.0)
Habits	12.0 (10.0-13.0)	12.0 (10.0-13.0)	11.0 (10.0-13.0)
Need and hunger	12.0 (11.0-14.0)	12.0 (11.0-14.0)	12.0 (10.0-13.0)
Health	12.0 (11.0-14.0)	12.0 (11.0-14.0)	13.0 (11.0-14.0)
Convenience	9.0 (7.0-11.0)	9.0 (7.0-11.0)	10.0 (8.0-11.0)
Pleasure	9.0 (7.0-11.0)	9.0 (8.0-11.0)	9.0 (7.0-10.0)
Traditional eating	6.0 (4.0-8.0)	6.0 (5.0-8.0)	5.0 (4.0-7.0)
Natural concerns	11.0 (9.0-12.0)	11.0 (9.0-12.0)	11.0 (8.5-12.0)
Sociability	7.0 (5.0-9.0)	7.0 (5.0-9.0)	6.0 (4.0-8.0)
Price	8.0 (6.0-10.0)	8.0 (6.0-10.0)	8.0 (6.0-10.0)
Visual appeal	6.0 (5.0-8.0)	6.0 (5.0-8.0)	5.0 (4.0-7.0)
Weight control	7.0 (5.0-9.0)	7.0 (5.0-9.0)	7.0 (5.0-9.0)
Affect regulation	5.0 (3.0-7.0)	5.0 (4.0-7.0)	4.0 (3.0-6.0)
Social norms	5.0 (4.0-7.0)	5.0 (4.0-7.0)	5.0 (4.0-7.0)
Social image	3.0 (3.0-4.0)	3.0 (3.0-5.0)	3.0 (3.0-4.0)
DEAS score	20 (18-25)	20 (18-25)	20 (17-23)
DEAS score category, No. (%)^b^			
<44.6	908 (94)	744 (93)	164 (96)
44.6-49.9	13 (1.3)	13 (1.6)	0
49.9-55.3	28 (2.9)	23 (2.9)	5 (2.9)
55.3-60.7	9 (0.9)	8 (1.0)	1 (0.6)
60.7-66.1	7 (0.7)	6 (0.8)	1 (0.6)
66.1-71.4	3 (0.3)	3 (0.4)	0
71.4-76.8	3 (0.3)	3 (0.4)	0

Abbreviation: DEAS, Disordered Eating Attitude Scale.

^a^ Scores range from 3 (minimum) to 15 (maximum).

^b^ Scores higher or equal to 66.1 identified individuals with disordered eating attitudes.

**Figure 3.**
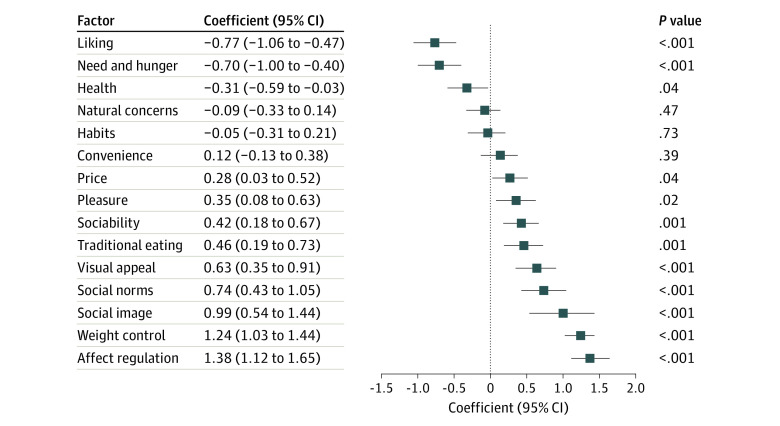
Associations Between Food Choice Motives and Disordered Eating Attitude Score by Multivariable Linear Regression Models

Multivariable linear regression models showed that “liking” (coefficient, −0.77; 95% CI, −1.06 to −0.47; *P* < .001), “need and hunger” (coefficient, −0.70; 95% CI, −1.00 to −0.40; *P* < .001) and “health” (coefficient, −0.31; 95% CI, −0.59 to −0.03; *P* = .04) were associated with lower disordered eating attitudes scores. In contrast, “price” (coefficient, 0.28; 95% CI, 0.03 to 0.52; *P* = .04), “pleasure” (coefficient, 0.35; 95% CI, 0.08 to 0.63; *P* = .02), “sociability” (coefficient, 0.42; 95% CI, 0.18 to 0.67; *P* = .001), “traditional eating” (coefficient, 0.46; 95% CI, 0.19 to 0.73; *P* = .001), “visual appeal” (coefficient, 0.63; 95% CI, 0.35 to 0.91; *P* < .001), “social norms” (coefficient, 0.74; 95% CI, 0.43 to 1.05; *P* < .001), “social image” (coefficient, 0.99; 95% CI, 0.54 to 1.44; *P* < .001), “weight control” (coefficient, 1.24; 95% CI, 1.03 to 1.44; *P* < .001), and “affect regulation” (coefficient, 1.38; 95% CI, 1.12 to 1.65; *P* < .001) were associated with higher disordered eating attitude scores (Figure 3).

## Discussion

The main findings of this survey of dietary behaviors among individuals who follow a vegan diet were that (1) the vast majority of participants (94%) were categorized into the lowest level of disordered eating attitudes, with only 0.6% of participants being identified with disordered eating attitudes; (2) the most important food choice motives were “need and hunger,” “liking,” “health,” “habits” and “natural concerns”; and (3) 9 food choice motives were associated with disordered eating attitudes.

Some studies have found that following a vegetarian diet may be associated with disordered eating attitudes and eating disorders.^4,5,6,31^ However, these associations are still controversial, with previous data showing no difference in disordered eating attitudes among vegetarians and omnivores.^11^ Interestingly, very low levels of disordered eating attitudes were found in our population. Namely, only 0.6% of our sample was identified with disordered eating attitudes. This is a significantly lower prevalence than that found in a similar Brazilian cohort within the general population (6.5%).^22^

These low rates could be partially explained by 62% of participants reporting “ethics and animal rights” and only 10% reporting “health reasons” as motivation to adhere to a vegan diet. Indeed, a previous small-scale study (62 participants) that comprised vegan dieters mainly due to ethical reasons did not find a higher risk of eating disorder symptoms.^32^ Moreover, eating motives commonly related to disordered eating attitudes (ie, weight control, affect regulation)^9,10^ were not reported as important eating motives in our population. In fact, vegetarians (including vegans) reported more eating motivations related to “health” and “natural content,” but less related to “weight control” when compared with omnivores.^11^ Conversely, another study exploring differences and commonalities in eating motives of vegetarians (including vegans) and omnivores found that motives were very similar across groups, with “liking” and “health” being among the top 4 motives, and “social norms,” “social image,” and “religion” being among the 4 least important ones.^33^ Likewise, findings of our study were that individuals who follow a vegan diet reported health-related motives as the most relevant, alongside with preference and physiological needs.

Contrary to our findings, previous research found that diet motives were not associated with greater disordered eating in a sample of 110 vegans.^7^ However, this was a smaller study, possibly precluding detection of the associations observed herein. In fact, it is known that some eating motives are related to disordered eating attitudes among the general population.^9,10^ Although we found that levels of disordered eating were low, we were able to identify certain food choice motives that were associated with increased DEAS scores in vegans: “sociability,” “traditional eating,” “visual appeal,” “pleasure,” “social norms,” “social image,” “weight control,” and “affect regulation.” This aligns with previous research that shows that individuals more concerned with their appearance and social image,^34,35,36^ as well as emotional eaters,^37^ exhibit more disordered eating attitudes, while individuals who respect internal cues to eat (eg, hunger, satiety, desires)^38,39,40^ and have a non–weight related health concern^9^ tend to have a better relationship with their own bodies and healthier eating attitudes. Although it is challenging to estimate the clinical significance of changes in DEAS score, the magnitude of association between some food choice motives found herein could be of potential clinical relevance, as an approximately 3 to 5 unit increase for some motives (ie, “social image,” “weight control” and “affect regulation”) would lead, on average, to a 5-point increase in DEAS score, shifting the respondent’s disordered eating attitudes category. As such, understanding food choice motives could be a clinically useful tool in identifying individuals at a higher risk of disordered eating behavior.

Our findings suggest that, although vegan diets could be associated with disordered eating attitudes,^4,5^ the presence of disordered eating attitudes in individuals following this diet may be associated with restriction itself (which could be generalized to any restrictive diet) and food choice motives (eg, weight control, affect regulation), rather than the decision to adopt a vegan diet, specifically. In fact, a study that reported higher levels of disordered eating behaviors in vegans compared with omnivores showed that it was significantly predicted by cognitive restraint and eating concern.^7^ Additionally, any claims that a vegan diet serves as a socially acceptable way to restrict food intake and conceal disordered eating attitudes should consider that such a disordered behavior is more likely among individuals whose eating choices, regardless of their diet type, are motivated by self-image and social acceptance. Therefore, it is unlikely that the association between vegan diet and disordered eating attitudes could be solely due to the diet per se, but to common factors related to the adherence to restrictive diets.

### Strengths and Limitations

Strengths of this study include the large sample size, specific dietary status (ie, only individuals who follow a vegan, but not a vegetarian, diet) and the use of validated questionnaires. In addition, our data add to the existing literature by identifying food choice motives that are associated with disordered eating attitudes in this population. It is important to highlight that DEAS evaluates disordered eating attitudes (ie, dysfunctional beliefs, thoughts, feelings, and relationship with food) rather than eating disorder symptoms.^22^ Nonetheless, although it is not possible to infer eating disorder prevalence from our data, assessment of disordered eating attitudes is of utmost value due to its prevalence and consequences.^41,42,43,44,45,46^

This study is not without limitations. First, the cross-sectional design does not allow causality to be inferred. Second, the use of self-reported weight and height may also be perceived as a limitation, although previous studies have shown that these self-reported measures are valid across different sexes and BMIs.^47,48^ Additionally, ours was a convenience sample, predominantly composed of female participants, with eutrophic BMI and high educational level (as compared with the average in the country), thus limiting our findings’ generalizability. Further studies with more heterogeneous, probabilistic samples are warranted, and so are studies including qualitative assessments, which could enable more in-depth understanding of disordered eating attitudes and food choice motives among individuals who follow a vegan diet.

## Conclusions

In conclusion, in this large survey among vegan dieters, we observed low levels of disordered eating attitudes. Moreover, “need and hunger,” “liking,” “health,” “habits,” and “natural concerns” were the most important food choice motives in this population. We also identified that several food choice motives were associated with disordered eating attitudes. Our findings may help shift the current perspective away from vegan diet per se leading to disordered eating, while highlighting the importance of food choice motives in general as factors associated with risk for disordered eating. Understanding food choice motives for shifting and adhering to diets that may impose restriction, which include, but are not limited to, vegan diets, may help tailor better interventions focused on promoting healthy eating and preventing or treating disordered eating.

## Notes

Supplement 1. **eTable. **Comparison of Subsets With Available and Missing Data Across Variables **eFigure 1. **Original Values and Imputed Values Across Iterations **eFigure 2. **Directed Acyclic Graph (DAG) With Possible Confounders for the Outcomes of Food Choice Motives on Disordered Eating Attitudes (1-9) **eFigure 3. **Associations Between Food Choice Motives and Disordered Eating Attitude Score by Multivariable Linear Regression Models, Using Complete Case Analysis (n = 650)
eReferences

Supplement 2.
Data Sharing Statement
