# Supplementary material for: Disordered Eating Attitudes and Food Choice Motives Among Individuals Who Follow a Vegan Diet in Brazil
Source: JAMA Netw Open. 2023 Jun 29;6(6):e2321065. doi: 10.1001/jamanetworkopen.2023.21065 (PMC10311387; doi:10.1001/jamanetworkopen.2023.21065)
Supplement: Supplement 1. — eTable. Comparison of Subsets With Available and Missing Data Across Variables eFigure 1. Original Values and Imputed Values Across Iterations eFigure 2. Directed Acyclic Graph (DAG) With Possible Confounders for the Outcomes of Food Choice Motives on Disordered Eating Attitudes (1-9) eFigure 3. Associations Between Food Choice Motives and Disordered Eating Attitude Score by Multivariable Linear Regression Models, Using Complete Case Analysis (n = 650) eReferences [file jamanetwopen-e2321065-s001.pdf]

Supplemental Online Content

Mazzolani BC, Smaira FI, Esteves GP, et al. Disordered eating attitudes and food choice motives among individuals who follow a vegan diet in Brazil. *JAMA Netw Open*. 2023;6(6):e2321065. doi:10.1001/jamanetworkopen.2023.21065

eTable. Comparison of Subsets With Available and Missing Data Across Variables

eFigure 1. Original Values and Imputed Values Across Iterations

eFigure 2. Directed Acyclic Graph (DAG) With Possible Confounders for the Outcomes of Food Choice Motives on Disordered Eating Attitudes (1-9)

eFigure 3. Associations Between Food Choice Motives and Disordered Eating Attitude Score by Multivariable Linear Regression Models, Using Complete Case Analysis (n = 650)

eReferences

This supplemental material has been provided by the authors to give readers additional information about their work.

© 2023 Mazzolani BC et al. *JAMA Network Open.*

eTable. Comparison of Subsets With Available and Missing Data Across Variables

Characteristic	**Overall**, N = 971[1]	**Subset with available data**, N = 650[1]	**Subset with missing data**, N = 321[1]
Age	29 (24, 36)	30 (24, 38)	28 (23, 35)
Sex			
Female	800 (82%)	528 (81%)	272 (85%)
Male	171 (18%)	122 (19%)	49 (15%)
Body weight (kg)	61 (54, 70)	61 (54, 70)	-
Height (cm)	165 (160, 170)	165 (160, 170)	-
BMI (g/kg²)	22.6 (20.3, 24.9)	22.6 (20.3, 24.9)	-
Educational level			
Postgraduate	354 (36%)	254 (39%)	100 (31%)
College education or technician, complete	289 (30%)	200 (31%)	89 (28%)
Undergoing college or technician education	223 (23%)	145 (22%)	78 (24%)
High school, completed	75 (7.7%)	39 (6.0%)	36 (11%)
High school, incomplete	22 (2.3%)	9 (1.4%)	13 (4.0%)
Elementary school, completed	3 (0.3%)	1 (0.2%)	2 (0.6%)
Elementary school, incomplete	5 (0.5%)	2 (0.3%)	3 (0.9%)
Other	1 (0.1%)	1 (0.1%)	0 (0%)
Socioeconomic class			
Class D/E	310 (32%)	195 (30%)	115 (36%)
Class C	252 (26%)	173 (27%)	79 (25%)
Class B	359 (37%)	255 (39%)	104 (32.7%)
Class A	50 (5.1%)	27 (4.2%)	23 (7.2%)
Smoking status	94 (9.7%)	58 (8.9%)	36 (11%)
Alcohol consumption			
No alcohol consumption	405 (42%)	269 (41%)	136 (42%)

© 2023 Mazzolani BC et al. *JAMA Network Open.*

Once to twice a month	180 (19%)	125 (19%)	55 (17%)
Twice to four times a month	273 (28%)	185 (28%)	88 (27%)
Twice to three times per week	100 (10%)	63 (9.7%)	37 (12%)
Four or more times per week	13 (1.3%)	8 (1.2%)	5 (1.6%)
Habitual physical exercise			
Does not exercise	194 (20%)	133 (20%)	61 (19%)
1-2 hour/week	211 (22%)	137 (21%)	74 (23%)
3-4 hour/week	143 (15%)	102 (16%)	41 (13%)
5-6 hour/week	204 (21%)	134 (21%)	70 (22%)
7 hour/week or more	219 (23%)	144 (22%)	75 (23%)
How long adhering to a vegan diet			
Less than one year	135 (14%)	81 (12%)	54 (17%)
1 to 2 years	224 (23%)	145 (22%)	79 (25%)
2 to 3 years	183 (19%)	123 (19%)	60 (19%)
3 to 4 years	149 (15%)	108 (17%)	41 (13%)
5 or more years	280 (29%)	193 (30%)	87 (27%)
Motivation for adhering to a vegan diet			
Ethics and animal rights	587 (60%)	386 (59%)	201 (63%)
Health reasons	103 (11%)	70 (11%)	33 (10%)
Environment	115 (12%)	80 (12%)	35 (11%)
Politics	41 (4.2%)	31 (4.8%)	10 (3.1%)
Life philosophy	111 (11%)	74 (11%)	37 (12%)
Medical restrictions	5 (0.5%)	3 (0.5%)	2 (0.6%)
Religion	2 (0.2%)	1 (0.2%)	1 (0.3%)
Sports performance	7 (0.7%)	5 (0.8%)	2 (0.6%)
Food choice motives			
Liking	12.00 (11.00-14.00)	12.00 (11.00-14.00)	12.00 (11.00-14.00)
Habits	12.00 (10.00-13.00)	12.00 (10.00-13.00)	12.00 (10.00-13.00)

© 2023 Mazzolani BC et al. *JAMA Network Open.*

Need and Hunger	12.00 (11.00-14.00)	12.00 (11.00-14.00)	12.00 (10.00-13.00)
Health	12.00 (11.00-14.00)	12.00 (11.00-14.00)	12.00 (10.00-14.00)
Convenience	9.00 (7.00-11.00)	9.00 (8.00-11.00)	9.00 (7.00-11.00)
Pleasure	9.00 (7.00-11.00)	9.00 (8.00-11.00)	9.00 (7.00-11.00)
Traditional Eating	6.00 (4.00-8.00)	6.00 (4.25-8.00)	6.00 (4.00-8.00)
Natural Concerns	11.00 (9.00-12.00)	11.00 (9.00-12.00)	11.00 (8.00-13.00)
Sociability	7.00 (5.00-9.00)	7.00 (5.00-9.00)	6.00 (5.00-8.00)
Price	8.00 (6.00-10.00)	8.00 (6.00-10.00)	8.00 (6.00-10.00)
Visual Appeal	6.00 (5.00-8.00)	6.00 (4.00-8.00)	6.00 (5.00-8.00)
Weight Control	7.00 (5.00-9.00)	7.00 (5.00-9.00)	7.00 (4.00-9.00)
Affect Regulation	5.00 (3.00-7.00)	5.00 (3.00-7.00)	5.00 (3.00-7.00)
Social Norms	5.00 (4.00-7.00)	5.00 (4.00-7.00)	5.00 (4.00-7.00)
Social Image	3.00 (3.00-4.00)	3.00 (3.00-4.00)	3.00 (3.00-4.00)
DEAS Score	20 (18-25)	20 (18-25)	20 (18-25)
DEAS score category			
< 44.6	908 (94%)	612 (94%)	296 (92%)
44.6 - 49.9	13 (1.3%)	6 (0.9%)	7 (2.2%)
49.9 - 55.3	28 (2.9%)	19 (2.9%)	9 (2.8%)
55.3 - 60.7	9 (0.9%)	7 (1.1%)	2 (0.6%)
60.7 - 66.1	7 (0.7%)	4 (0.6%)	3 (0.9%)
66.1 - 71.4*	3 (0.3%)	1 (0.2%)	2 (0.6%)
71.4 - 76.8	3 (0.3%)	1 (0.2%)	2 (0.6%)

[1] Median (IQR); n (%)

© 2023 Mazzolani BC et al. *JAMA Network Open.*

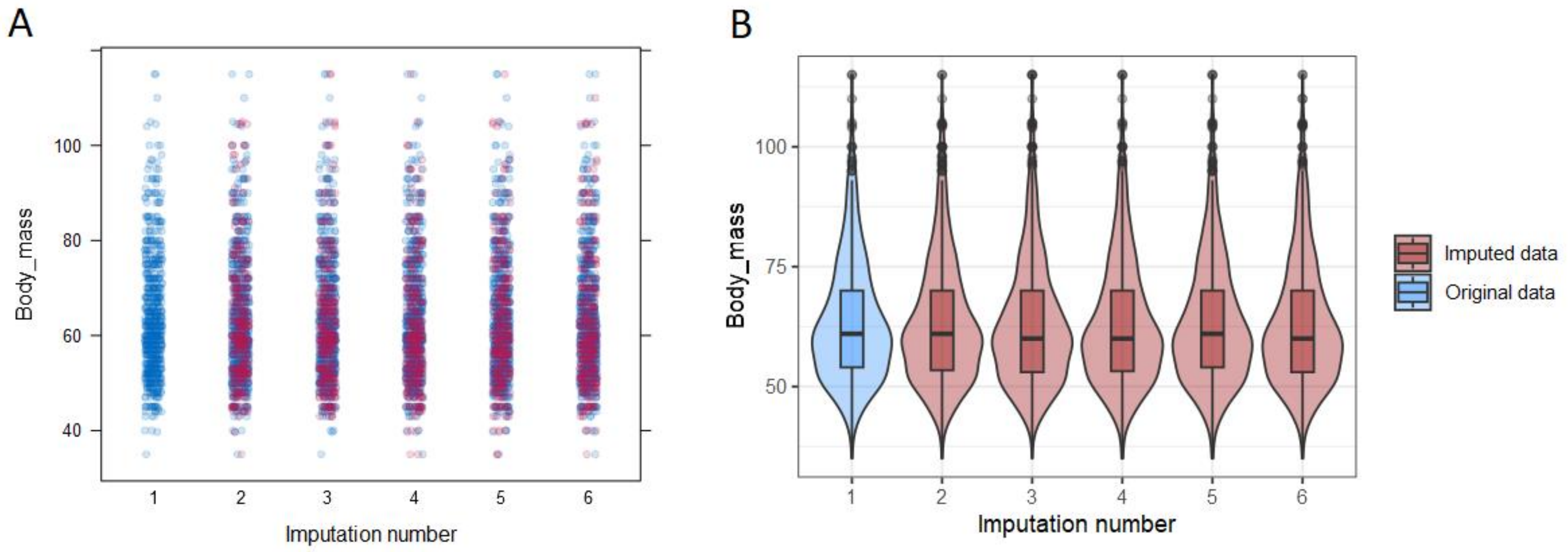

eFigure 1. Original Values and Imputed Values Across Iterations

A) Strip plot showing original values (blue dots) and imputed values (red) across iterations (1 = initial data, 6 = 5th imputation iteration) of the body mass variable. B) Boxplot and violin plots showing summary statistics and distribution of original and imputed data. Plots indicate good performance of the imputation method, with similar values of the imputed and original of body mass across iterations.

© 2023 Mazzolani BC et al. *JAMA Network Open.*

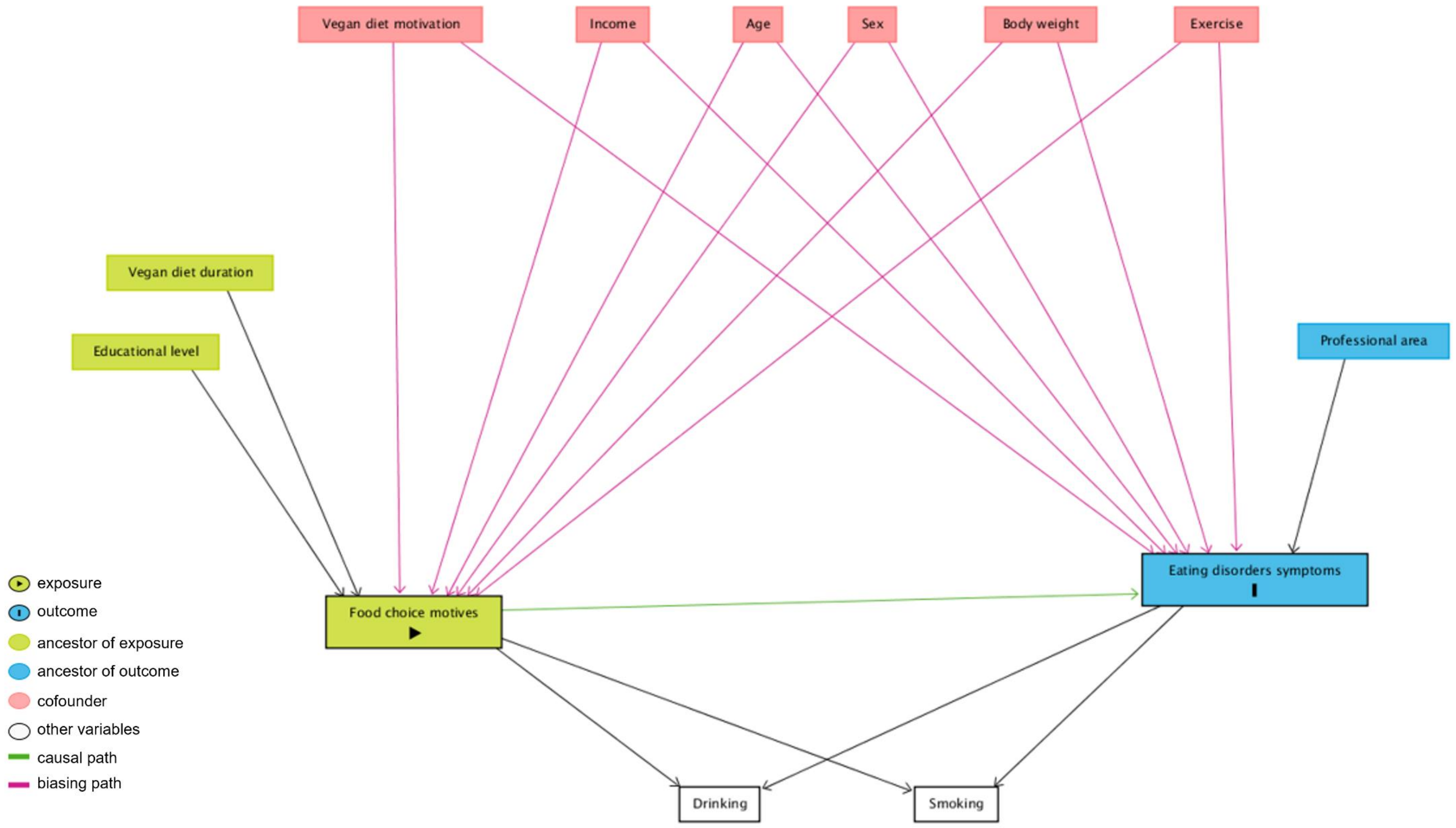

eFigure 2. Directed Acyclic Graph (DAG) With Possible Confounders for the Effects of Food Choice Motives on Disordered Eating Attitudes (1-9)

© 2023 Mazzolani BC et al. *JAMA Network Open.*

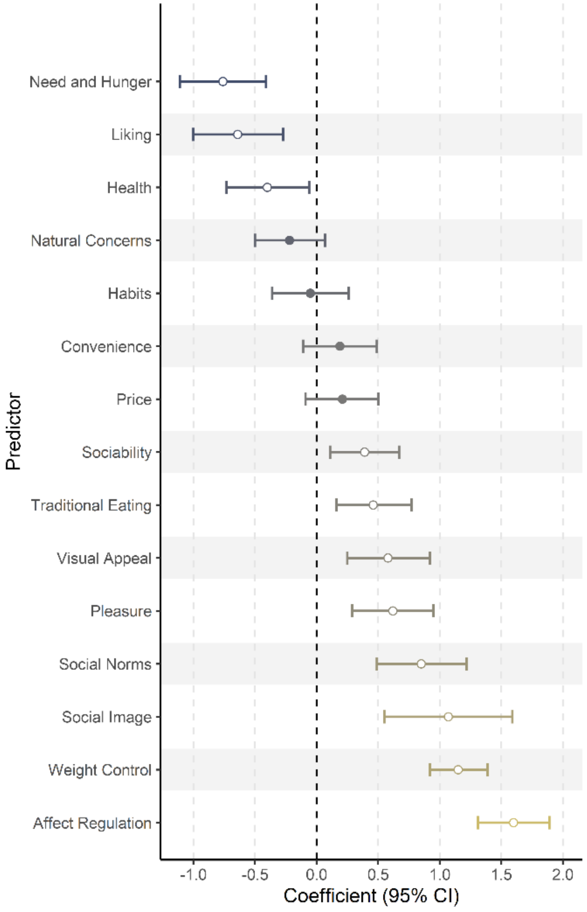

Coefficient (95% CI)	p-value
-0.76 (-1.11; -0.41)	<0.0001
-0.64 (-1.00; -0.27)	<0.001
-0.40 (-0.73; -0.06)	0.02
-0.22 (-0.50; 0.07)	0.14
-0.05 (-0.36; 0.26)	0.74
0.19 (-0.11; 0.49)	0.21
0.21 (-0.09; 0.50)	0.17
0.39 (0.11; 0.67)	<0.01
0.46 (0.16; 0.77)	<0.01
0.58 (0.25; 0.92)	<0.001
0.62 (0.29; 0.95)	<0.001
0.85 (0.49; 1.22)	<0.0001
1.07 (0.55; 1.59)	<0.0001
1.15 (0.92; 1.39)	<0.0001
1.60 (1.31; 1.89)	<0.0001

eFigure 3. Associations Between Food Choice Motives and Disordered Eating Attitude Score by Multivariable Linear Regression Models, Utilizing Complete Case Analysis (n = 650)

© 2023 Mazzolani BC et al. *JAMA Network Open.*

eReferences

1. Silva WRD, Santana MS, Maroco J, Maloa BFS, Campos J. Body weight concerns: Cross-national study and identification of factors related to eating disorders. PloS one. 2017;12(7):e0180125.
2. Fekih-Romdhane F, Daher-Nashif S, Alhuwailah AH, Al Gahtani HMS, Hubail SA, Shuwiekh HAM, et al. The prevalence of feeding and eating disorders symptomology in medical students: an updated systematic review, meta-analysis, and meta-regression. Eating and weight disorders : EWD. 2022;27(6):1991-2010.
3. Fuller-Tyszkiewicz M, Rodgers RF, Maïano C, Mellor D, Sicilia A, Markey CH, et al. Testing of a model for risk factors for eating disorders and higher weight among emerging adults: Baseline evaluation. Body image. 2022;40:322-39.
4. Koch SV, Larsen JT, Plessen KJ, Thornton LM, Bulik CM, Petersen LV. Associations between parental socioeconomic-, family-, and sibling status and risk of eating disorders in offspring in a Danish national female cohort. The International journal of eating disorders. 2022;55(8):1130-42.
5. Konttinen H, Halmesvaara O, Fogelholm M, Saarijärvi H, Nevalainen J, Erkkola M. Sociodemographic differences in motives for food selection: results from the LoCard cross-sectional survey. International Journal of Behavioral Nutrition and Physical Activity. 2021;18(1):71.
6. Mahn HM, Lordly D. A Review of Eating Disorders and Disordered Eating amongst Nutrition Students and Dietetic Professionals. Canadian journal of dietetic practice and research : a publication of Dietitians of Canada = Revue canadienne de la pratique et de la recherche en dietetique : une publication des Dietetistes du Canada. 2015;76(1):38-43.
7. Moraes JMM, Moraes CHC, Souza AAL, Alvarenga MDS. Food choice motives among two disparate socioeconomic groups in Brazil. Appetite. 2020;155:104790.
8. Stice E, Gau JM, Rohde P, Shaw H. Risk factors that predict future onset of each DSM-5 eating disorder: Predictive specificity in high-risk adolescent females. Journal of abnormal psychology. 2017;126(1):38-51.
9. Stice E, Desjardins CD. Interactions between risk factors in the prediction of onset of eating disorders: Exploratory hypothesis generating analyses. Behaviour research and therapy. 2018;105:52-62.

© 2023 Mazzolani BC et al. *JAMA Network Open.*
