# Supplementary material for: Disordered Eating Attitudes and Food Choice Motives Among Individuals Who Follow a Vegan Diet in Brazil
Source: JAMA Netw Open. 2023 Jun 29;6(6):e2321065. doi: 10.1001/jamanetworkopen.2023.21065 (PMC10311387; doi:10.1001/jamanetworkopen.2023.21065)
Supplement: Supplement 2. — Data Sharing Statement [file jamanetwopen-e2321065-s002.pdf]

Data Sharing Statement

Mazzolani. Disordered Eating Attitudes and Food Choice Motives Among Individuals Who Follow a Vegan Diet in Brazil. *JAMA Netw Open*. Published June 29, 2023. doi:10.1001/jamanetworkopen.2023.21065

Data
Data available: Yes
Data types: Deidentified participant data
How to access data: hars@usp.br
When available: With publication

Supporting Documents
Document types: Statistical/analytic code
How to access documents: hars@usp.br
When available: With publication

Additional Information
Who can access the data: Researchers whose proposed use of the data has been approved
Types of analyses: For specified purpose
Mechanisms of data availability: With investigator support
